# Live Imaging of embryogenic structures in *Brassica napus* microspore embryo cultures highlights the developmental plasticity of induced totipotent cells

**DOI:** 10.1007/s00497-020-00391-z

**Published:** 2020-07-10

**Authors:** Patricia Corral-Martínez, Charlotte Siemons, Anneke Horstman, Gerco C. Angenent, Norbert de Ruijter, Kim Boutilier

**Affiliations:** 1 Plant Development Systems, Wageningen University and Research, P.O. Box 16, 6700 AA Wageningen, The Netherlands; 2 Laboratory of Molecular Biology, Wageningen University and Research, P.O. Box 633, 6700 AP Wageningen, The Netherlands; 3 Laboratory of Cell Biology, Wageningen University and Research, P.O. Box 633, 6700 AP Wageningen, The Netherlands; 4 Wageningen Light Microscopy Centre, Wageningen University and Research, P.O. Box 633, 6700 AP Wageningen, The Netherlands; 5 Present Address: Cell Biology Group, COMAV Institute, Universitat Politècnica de València (UPV), Camino de Vera, s/n. 46022, València, Spain

**Keywords:** *Brassica napus*, *LEAFY COTYLEDON1*, Microspore embryogenesis, Suspensor, Time-lapse imaging, Totipotency

## Abstract

*****Key message***:**

**In vitro embryo development is highly plastic; embryo cell fate can be re-established in tissue culture through different pathways.**

**Abstract:**

In most angiosperms, embryo development from the single-celled zygote follows a defined pattern of cell divisions in which apical (embryo proper) and basal (root and suspensor) cell fates are established within the first cell divisions. By contrast, embryos that are induced in vitro in the absence of fertilization show a less regular initial cell division pattern yet develop into histodifferentiated embryos that can be converted into seedlings. We used the *Brassica napus* microspore embryogenesis system, in which the male gametophyte is reprogrammed in vitro to form haploid embryos, to identify the developmental fates of the different types of embryogenic structures found in culture. Using time-lapse imaging of *LEAFY COTYLEDON1*-expressing cells, we show that embryogenic cell clusters with very different morphologies are able to form haploid embryos. The timing of surrounding pollen wall (exine) rupture is a major determinant of cell fate in these clusters, with early exine rupture leading to the formation of suspensor-bearing embryos and late rupture to suspensorless embryos. In addition, we show that embryogenic callus, which develops into suspensor-bearing embryos, initially expresses transcripts associated with both basal- and apical-embryo cell fates, suggesting that these two cell fates are fixed later in development. This study reveals the inherent plasticity of in vitro embryo development and identifies new pathways by which embryo cell fate can be established.

**Electronic supplementary material:**

The online version of this article (10.1007/s00497-020-00391-z) contains supplementary material, which is available to authorized users.

## Introduction

Plant embryogenesis begins with formation of a totipotent zygote that develops after fusion of the male and female gametes. Embryo development in angiosperms, as represented by the model dicot plant *Arabidopsis thaliana* (arabidopsis), can be highly regular and proceeds through defined morphological stages and developmental phases. In arabidopsis, the first zygote division is asymmetric, resulting in a smaller apical cell and larger basal cell. The apical cell is the progenitor of the embryo proper, while the basal cell forms the future root pole and the suspensor, an ephemeral multicellular uniseriate structure that functions in nutrient and hormone pathways. The establishment of an apical-basal axis is a common theme in both monocot and dicot embryo development, although the regularity of the cell division pattern and the timing and presence of key developmental processes often differ greatly between plant species (Kaplan and Cooke 1997; Radoeva and Weijers 2014; Zhao et al. 2017).

Many plant cells are able to form embryos in the absence of fertilization, either in vivo as part of an alternative asexual reproduction pathway or in vitro in response to inducer treatments (Vijverberg et al. 2019; Méndez-Hernández et al. 2019; Testillano 2019). Microspore embryogenesis (ME) is a form of in vitro totipotency in which cultured immature male haploid gametophytes (microspores and pollen) are induced to form embryos, usually in response to a stress treatment (Soriano et al. 2013; Testillano 2019). Haploid embryos develop in vitro from single cells, most commonly from the unicellular microspore or from the vegetative cell of bicellular pollen (Sunderland 1974; de F. Maraschin et al. 2008; Daghma et al. 2014). The predominately single cell origin of microspore embryos makes ME a tractable system to study embryo development in the absence of parental and filial tissues. *Brassica napus* is a well-studied model system for ME, in part due to the large number of responding genotypes that differ in the extent to which they are able to form haploid embryos (Bhowmik et al. 2011). In *B. napus* ME has been used to study various aspects of (in vitro) embryo development, including totipotency (Joosen et al. 2007; Malik et al. 2007; Li et al. 2014), cell wall architecture (El-Tantawy et al. 2013; Solís et al. 2016; Corral-Martínez et al. 2019; Rivas-Sendra et al. 2019), hormone signalling (Hays 2000; Dubas et al. 2013, 2014; Soriano et al. 2014; Robert et al. 2015; Rodríguez-Sanz et al. 2015), and the role of the suspensor in patterning the embryo proper (Supena et al. 2008; Soriano et al. 2014).

A heat stress treatment is used to induce ME in *B. napus*. After the heat stress treatment, the vast majority of cultured cells either stop dividing or continue gametophytic development, while only a small proportion of the original population is induced toward embryo development. Three types of embryogenic structures have been identified within the first week of culture: compact embryos that lack a suspensor, suspensors and/or suspensors with a few-celled embryo proper, and different types of callus-like structures (Telmer et al. 1995; Ilić-Grubor et al. 1998; Supena et al. 2008; Soriano et al. 2014; Li et al. 2014). At this time, compact embryos are still enclosed by the pollen wall (exine), while suspensors/suspensor-bearing embryos and embryogenic callus already show different degrees of exine rupture. The frequency and quality of suspensor-bearing embryo formation depends on the genotype and the culture conditions. For example, a high proportion of embryos with a long uniseriate suspensor filament can be induced in some genotypes (Supena et al. 2008), while only short suspensors, often with abnormal cell divisions, are induced in others (Soriano et al. 2014). Callus-like structures were initially thought to represent a non-embryogenic type of growth, based on the accumulation of starch and/or lipids (pollen characteristics), the presence of thick cell walls and reduced cell adhesion (Telmer et al. 1995; Ilić-Grubor et al. 1998), but were later shown to be embryogenic based on their expression of multiple embryo identity genes (Li et al. 2014). It has been suggested that both exine-enclosed and suspensor-bearing embryos develop further into histodifferentiated embryos (Telmer et al. 1995; Yeung et al. 1996; Nitta et al. 1997; Supena et al. 2008; Tang et al. 2013), while the fate of embryogenic callus is not known.

Changes in the chromatin landscape have been observed during the course of ME, and application of epidrugs that target chromatin-modifying enzymes influences the induction and progression of haploid embryogenesis (Solís et al. 2012; El-Tantawy et al. 2014; Rodríguez-Sanz et al. 2014; Li et al. 2014; Pandey et al. 2017; Berenguer et al. 2017), suggesting endogenous roles for chromatin-modifying proteins during ME. In *B. napus*, application of the histone/lysine deacetylase (HDAC/KDAC) inhibitor trichostatin A (TSA) enhances the production of differentiated embryos and embryogenic callus when applied alone or together with heat stress (Li et al. 2014). The morphological similarities in developmental response between heat-stressed and heat stress plus TSA-treated cultures (HS + TSA), together with the observation that TSA treatment enhances embryo production in ME culture systems induced by different stresses (Zhang et al. 2016; Pandey et al. 2017; Jiang et al. 2017), suggest that increased histone/protein acetylation is a conserved component of totipotency induction in the male gametophyte.

Here we used time-lapse imaging to determine the fate of embryogenic callus in *B. napus* microspore culture. We show that embryogenic callus, despite its poor cell morphology and low growth potential, develops into suspensor-bearing embryos. Different pathways to suspensor-bearing embryo development from embryogenic callus could be defined based on the orientation of the first cell division, the extent of exine rupture, and the degree of cell adhesion. This newly discovered route to haploid embryo development highlights the high degree of developmental plasticity found in haploid embryo cultures.

## Materials and methods

### Plant material and culture

The *Brassica napus* L. DH12075 genotype was used for haploid embryo culture. Plants were grown and cultured as in Li et al. (2014). The *LEC1:LEC1-GFP* reporter line was previously described (Soriano et al. 2014; Li et al. 2014). Trichostatin A (TSA) was dissolved in DMSO (Sigma) and applied as described below.

### Sample preparation for light microscopy

Whole-mount samples for light microscopy were collected by centrifugation and fixed overnight at 4 °C with 4% paraformaldehyde in PBS (pH 7.4) before being washed three times with PBS and stored at 4 °C in 0.1% paraformaldehyde in PBS until use.

Samples prepared for a separate transmission electron microscopy study were also used for light microscopy. Samples were centrifuged, fixed in Karnovsky solution (Karnovsky 1965), and then post-fixed with 2% OsO_4_. The buffer was replaced with one to two drops of warm liquid gelatin (15%) to immobilize and concentrate the samples. The samples were centrifuged (1 min at 8000 rpm), cooled on ice for gelatin solidification and then incubated overnight at 4 °C with 20 µl of 1% paraformaldehyde to harden the gelatin. Gelatin-embedded samples were cut in small pieces and kept in buffer until use. All of the above solutions were prepared in cacodylate buffer (Hayat 1981). Samples were dehydrated in a progressive ethanol series and embedded and polymerized in Embed-812 resin (Electron Microscopy Science). One micron-thick sections were made with a Leica UC6 ultramicrotome. At least three blocks were used for each treatment, and at least 30 to 50 embryogenic structures were analyzed per block.

### Light and confocal microscopy

Pectins were stained in whole mounts with Ruthenium Red (500 mg/L; Merck Sigma-Aldrich) dissolved in PBS (Luft 1971). The whole mounts were stained for 10 min and then observed using a Nikon Eclipse E1000 microscope. At least 100 embryogenic structures were examined per treatment. Cell walls were stained by a 30 min incubation in 0.1% (v/v) SCRI Renaissance 2200 (Musielak et al. 2015) prior to observation. Staining of cytoplasmic membranes was performed directly in live samples using CellBrite™ Orange (Biotium) (200 × dilution; 20 min. incubation). Nuclei were stained with 1 μg/ml DAPI as in Custers et al. (Custers et al. 1994). Cell viability was assessed using fluorescein diacetate (FDA) and propidium iodide (PI) (Merck Sigma-Aldrich) as in Ibidi Application Note 33.

Fluorescence was observed with Leica SP5 confocal laser scanning microscope. SR2200 and DAPI were excited with a 405-nm laser line and emission recorded respectively, between 415–476 and 458–487 nm. GFP and FDA were excited with the 488-nm laser line and emission detected between 510–530 nm and 535, respectively. CellBrite Orange and PI were excited with a 561 nm laser line and emission recorded respectively, between 565–636 nm and 617–640 nm. Images were processed with Leica Application Suite Advanced Fluorescence (LAS AF) and FIJI software. At least 100 embryogenic structures were examined per treatment.

To measure cell wall thickness and the distance between adjacent cell membranes, the SCRI Renaissance 2200 and CellBrite™ Orange images were first transformed from RGB format to 16-bit images for analysis in Image J. The scale was adjusted for the different image magnifications. Twenty different structures were measured per category, and in each structure, the width of the cell wall or distance between adjacent plasma membranes was measured at five different points to obtain 100 measurements.

### Field emission scanning electron microscopy (FESEM)

Material from 5-day-old cultures was collected by centrifugation, fixed in Karnovsky solution and then washed three times with 0.025 M cacodylate. Post-fixation in 1% OsO_4_ solution (60 min., RT) was performed one day before imaging, followed by three washes in distilled H_2_O. The samples were then dehydrated in a progressive ethanol series and stored overnight in absolute ethanol. The next day the samples were transferred twice to absolute ethanol for 60 min. each. Critical point drying was performed according to Robards and Wilson (1993). Imaging was performed using a Zeiss Ultra 55 electron microscope.

### Time lapse imaging

Isolated microspores (4 × 10^5^ microspores /ml) were cultured with 0.05 µM TSA for 24 h at 33 °C (HS) in 15-ml plastic tubes (Greiner; 9 ml/tube), after which the TSA was removed by centrifugation in a cooled centrifuge at 130 g and replaced with fresh liquid NLN-13 medium. The culture density was re-adjusted to 4 × 10^5^ microspores per/ml, and then the samples were cultured for an additional four days at 25 °C in 6-cm plates before embedding in agarose. Prior to embedding, the microspore density was increased to 1.0 × 10^6^ microspores/ml to facilitate imaging. Low melting (26–30 °C) agarose (SeaPlaque, Duchefa) was heated in a microwave until liquid and stored in microcentrifuge tubes in a 33 °C block heater until use. Double-strength NLN-13 medium was warmed to 33 °C and stored in microcentrifuge tubes in a 33 °C block heater until use. Petri dishes with a 35-mm grid (Ibidi, catalogue no: 80156) were pre-warmed to 33 °C on an electric heater plate. Equal volumes of the microspore culture, double-strength NLN-13 medium, and low melting agarose were mixed thoroughly in a microcentrifuge tube and placed in a 33 °C block heater. A drop of 20 µl of the above mixture was pipetted into the pre-warmed Petri dish and then spread very quickly to cover the bottom of the dish. The layer was allowed to gel for 10 min by placing the Petri dish on ice without the lid (to prevent condensation). 200–300 µl of NLN-13 culture medium was pipetted slowly along the inner side of the Petri dish to cover the agarose.

Imaging was performed at 25 °C in a temperature-controlled room using a Yokogawa CSU-X1 spinning disk confocal microscope (Andor Revolution XDi) on a Nikon Eclipse Ti microscope. Morphology and LEC1-GFP expression were used to preselect embryogenic cell clusters for tracking. GFP was detected using a 488 nm laser passing a 486–491 BP excitation, and a 500–550 BP emission filter. Imaging was performed with 20× PlanApo VC (NA 0.75) or 60× Plan Fluor (NA 1.4, oil immersion) objective on an Andor iXon888 EMCCD camera. The position of embryogenic structures on the dish was recorded and tracked for up to five days using the motorized xyz- piezo accuracy stage (ASI-150, Eugene-Oregon, USA) and Perfect Focus System (PFS3, Nikon) to prevent drift. Depending on the experiment, photographs for each structure were taken every 4, 6, 12 and/or 24 h. In some experiments, a single image was acquired for each structure and in others six-image z-stacks were made. Image acquisition was controlled in the Multi-Dimensional Acquisition mode of MetaMorph 7.8.3.0 (Molecular Devices) imaging software. The imaging was performed in the dark in a temperature-controlled stage and room (25 °C). An adjustable stage-top incubator (Tokai-hit) was used to warm the cover at 28 °C above RT to prevent condensation on the lid of the petri dish.

### Samples for transcriptome analysis

The following samples were collected for gene expression analysis: day 0, freshly isolated culture; day 2 HS, culture treated with HS (33 °C) for 1 day, followed by medium refreshment and incubation at 25 ˚C for 1 day; day 4 HS, culture treated with HS (33 °C) for 1 day, followed by medium refreshment and incubation at 25 °C for 3 days; day 2 HS + 0.5 µM TSA, culture treated with HS (33 °C) + 0.5 µM TSA for 1 day, followed by medium refreshment without TSA and incubation at 25 ˚C for 1 day; day 4 HS + 0.5 µM TSA, culture treated with HS (33 °C) + 0.5 µM TSA for 1 day, followed by medium refreshment without TSA and incubation at 25 °C for 3 days; globular embryos, purified globular embryos from day 8 HS + 0.05 µM TSA-treated cultures. For this sample, cultures were treated with HS (33 °C) + 0.05 µM TSA for 1 day, followed by medium refreshment without TSA and incubation at 25 °C for 7 days, followed by sieving through a 50-µm nylon mesh to enrich for globular-stage embryos.

### RNA sequencing and data analysis

Each sample was derived from pooled material, corresponding to 400,000 to 1,000,000 cells at the start of culture. The cultures were centrifuged, the medium removed, and the pellet frozen in liquid nitrogen. The pelleted material was disrupted with stainless steel beads and the RNA was extracted using the InviTrap Spin Plant RNA Mini Kit (Stratec). Two micrograms of DNAse-treated RNA was used as the template for RNA-seq library preparation (TruSeq RNA Library Prep Kit). Barcoded libraries were pooled and sequenced on a HiSeq2500 platform. Raw reads were trimmed by removing adapter sequences before mapping of the reads to the *B. napus* genome.

The RNA sequencing data can be retrieved from Gene Expression Omnibus database under accession number GSE140969. 125 nt paired-end reads were mapped to *B. napus* genome version 4.1 (https://brassicadb.org) using the RNA sequencing analysis package CLC Genomics Workbench 11 (www.clcbio.com) with the following parameters: min. read length fraction = 0.8, min. read similarity fraction = 0.8, mismatch cost = 2, insertion cost = 3, deletion cost = 3, strand specific = both, maximum number of hits for a read = 10, count paired reads as two = no. The R-Bioconductor package Limma-Voom (Law et al. 2014) was used to normalize the reads over the entire experiment. The trimmed mean of M-values (TMM) normalization method (Robinson and Oshlack 2010) was used to account for small biases in each sample’s overall read library size. Transcripts with expression levels greater than three counts per million (CPM) in at least three libraries were retained.

Principal component analysis (PCA) was performed with the R function *prcomp (center* = *TRUE, scale* = *FALSE).* Pearson correlation of log2(CPM) values was calculated between the different treatments using the *gg_scatter* function (from ggpubr package) in R. A heat map was created with the R function *heatmap.2* using log2(CPM) values.

The *B. napus* gene identifiers and the corresponding expression values for the heat map are listed in Supplemental Data File 1. The Integrative Orthology Viewer (PLAZA4.0 database, https://bioinformatics.psb.ugent.be/plaza/versions/plaza_v4_dicots/) was used to identify orthologues of *Arabidopsis thaliana* genes in *B. oleracea* and *B. rapa*, the parent genomes of amphidiploid *B. napus*. The gene sequences retrieved from the PLAZA4.0 database were BLASTed against *B. napus* cDNAs (NCBI) to identify the most similar *B. napus* genes.

## Results

### Embryogenic cell types in microspore embryo culture

**Fig. 1:**
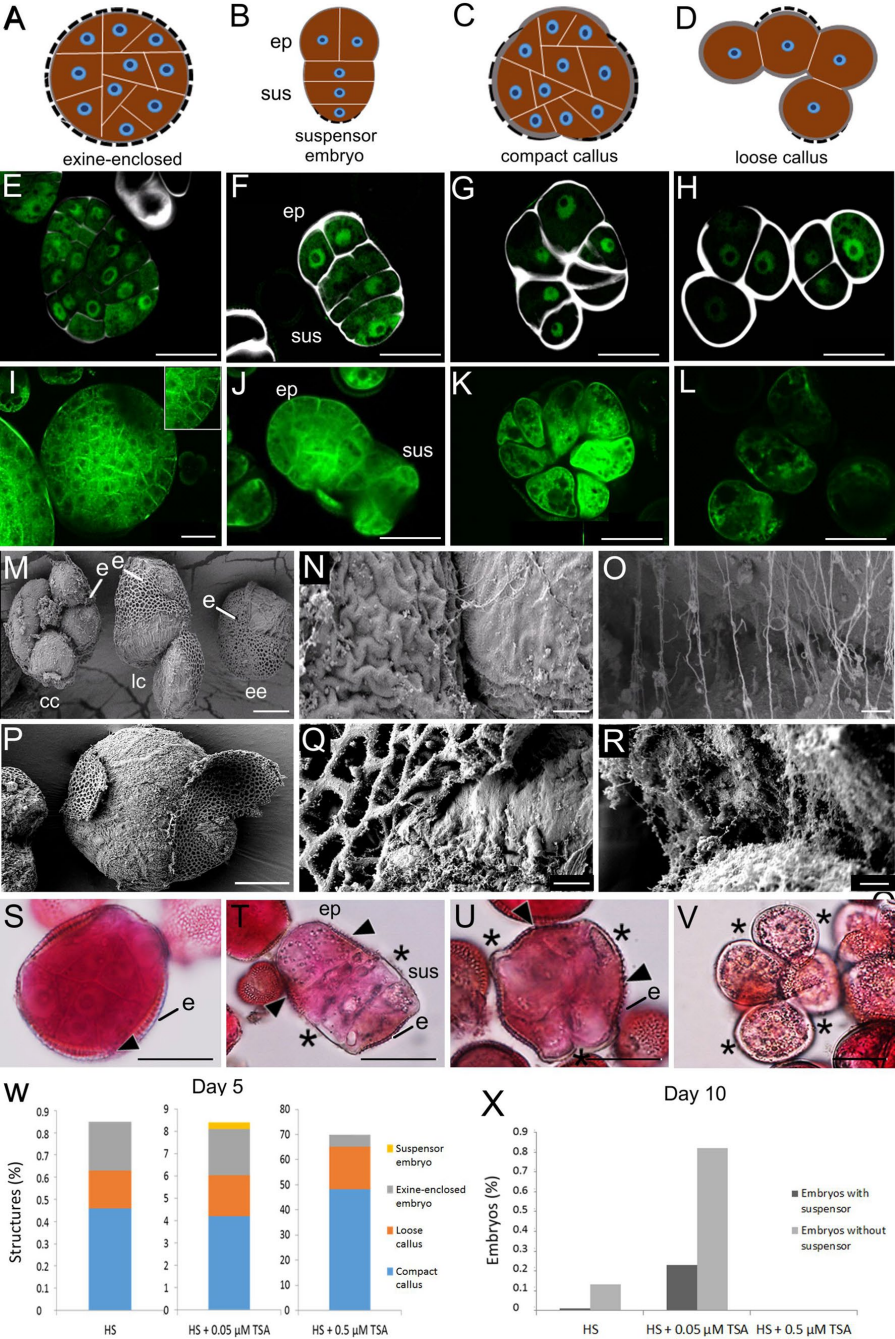
Embryogenic structures found in *B. napus* microspore cultures. **a–d** Schematic representation of the different types of embryogenic structures found in 5-day-old cultures. Dotted line, exine; ep, embryo proper; s, suspensor. **e**–**h** CLSM images of *LEC1:LEC1-GFP* expressing (green) structures counter-stained with the SCRI Renaissance 2200 cell wall stain (white). *LEC1:LEC1-GFP* expression marks embryogenic cells. **e** compact exine-enclosed embryo; **f** suspensor-bearing embryo. **g** compact callus. **h** loose callus. Labels: sus, suspensor; ep, embryo proper. **i–l** CLSM images of Cell Brite-stained (green) membranes of **i**, an exine-enclosed embryo; **j.** a suspensor-bearing embryo; **k** compact embryogenic callus. **l** loose embryogenic callus Labels: sus, suspensor; ep, embryo proper. **m–r** FESEM images of embryogenic structures. **m** Three different types of embryogenic structures. **n** Detail of the area between two cells of a callus-like structure where the exine has ruptured; **o** Same area in **n**. with higher magnification, showing that only a few fibers connect adjacent cells. **p** Exine-enclosed embryo where the exine has started to rupture. **q** Detail of the exine rupture site in an exine-enclosed embryo. **r** Same area in **q**, with higher maginification, showing that many fibers connect adjacent cells. Labels: ee, exine-enclosed embryo; cc, compact callus; lc, loose callus; e, exine. **s–v** Pectin deposition in embryogenic structures. Whole-mount samples stained with Ruthenium Red. **s**, exine-enclosed embryo; **t**, suspensor-bearing embryo; **u**, compact callus; **v**, loose callus. Label: e, exine; sus, suspensor; ep, embryo proper; arrowhead, area with pectin staining; asterisk, area without pectin staining. **w**. Proportion of different embryogenic structures found in 5-day old cultures induced by heat stress (HS), a combined heat stress and 0.05 µM TSA treatment and a combined heat stress and 0.5 µM TSA treatment. **x** Proportion of histodifferentiated embryos with or without a suspensor in 10-day-old cultures found in the different culture treatments described in (**w**). ‘Suspensor embryo’ refers to both suspensors and suspensor-bearing embryos, i.e., a suspensor with an embryo proper. Scale bars: **a–d** 10 μm; **e–l** 25 μm; **m** 10 μm; **n** 1 μm; **o** 300 nm; **p** 10 μm; **q** 1 μm; **r** 300 nm; **s–v**, 25 μm

Microspore embryo cultures from the *B. napus* genotype DH12075 have a low histodifferentiated embryo yield and a high proportion of embryogenic callus (Soriano et al. 2014; Li et al. 2014). As such, DH12075 is a suitable genotype for understanding the different factors that contribute to recalcitrance for ME. DH12075 haploid embryo cultures are induced by heat stress (HS; 33 °C) from isolates with a high proportion of mid-uninucleate microspores (Supplemental Fig. 1). After 5 days of culture, only a low percentage of the male gametophytes have switched to embryogenic growth in response to the initial HS treatment (Fig. 1w). Three types of embryogenic structures are observed at this time: (1) exine-enclosed embryos; (2) suspensors or suspensors-bearing embryos, 3) and two types of embryogenic callus, termed compact and loose callus (Fig. 1a–h, t). Only a small proportion of these embryogenic structures will eventually form differentiated embryos (Fig. 1x).

At day 5 of culture, exine-enclosed embryos comprise a globular mass of compact cells without any obvious morphological signs of histodifferentiation. Thereafter, the increasing volume of the dividing embryo eventually stretches and weakens the surrounding exine so that it breaks and releases the embryo. Differentiation of the embryo commences with exine rupture (Hause et al. 1994; Yeung et al. 1996; Nitta et al. 1997; Soriano et al. 2014). Most exine-enclosed embryos do not have a suspensor, but when present, it is visible as a small protrusion after exine rupture.

Suspensors or suspensor-bearing embryos are rarely seen at day 5 of culture in HS-treated DH12075 (Soriano et al. 2014). When present, their morphology is highly variable, ranging from short protrusions to filaments of different lengths, most of which show ectopic cell divisions (Fig, 1F, Supplemental Fig. 2). Suspensors or suspensor-bearing embryos present at day 5 of culture most likely develop ab initio from a single microspore/pollen in which the surrounding exine is no longer intact, as described in Supena et al. (2008).

**Fig. 2:**
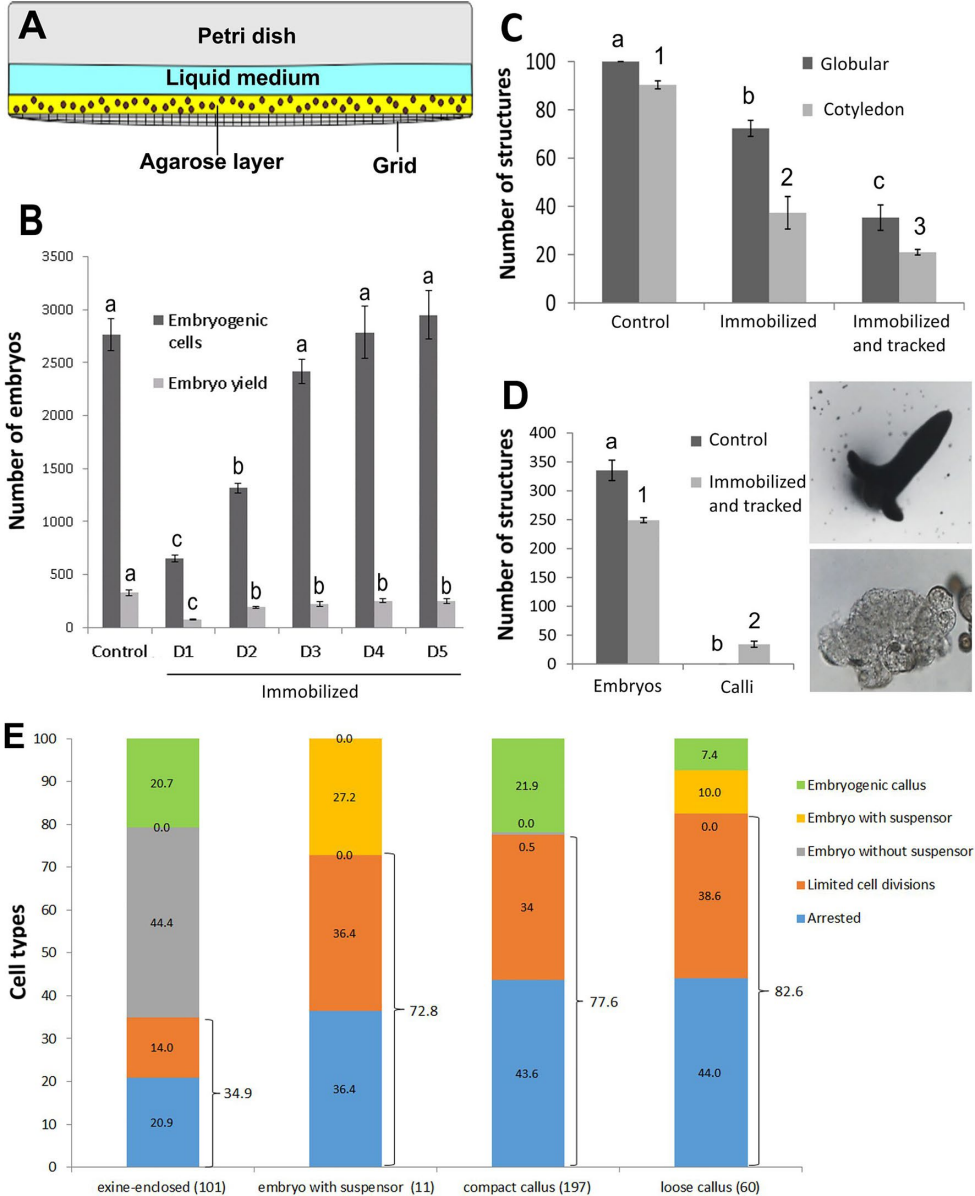
Time-lapse imaging of *B. napus* microspore embryo cultures. **a** The immobilization system makes use of a gridded Petri dish with a coverslip-like bottom, an agarose layer with at most two layers of cells, and a layer of liquid medium. The effect of immobilization on embryogenic cell development and differentiated embryo yield. Shown are the number of divided embryogenic cells and embryo yield per Petri dish without immobilization (control) and after immobilization on different days (D) after the start of culture. Embryogenic cells were counted on day 5- and differentiated embryos on day 14 of culture. **c** The effect of immobilization and tracking on the speed of embryo development. Cultures were immobilized on day 5 of culture, tracked for 3 days and then cultured further without tracking. The number of globular embryos/Petri dish was determined immediately after tracking (day 8), and the number of cotyledon-stage embryos/Petri dish was determined six days after the tracking period ended (day 14). Control cultures were neither immobilized nor tracked. **d** The number of embryos and calli per Petri dish on day 14 in control (not immobilized, not tracked) and immobilized and tracked cultures. For the latter, cultures were immobilized on day 5, tracked for three days, and then cultured further without tracking until day 14 of culture. A typical cotyledon-stage embryo (top panel) and a large callus structure (bottom panel) that develop after immobilization and tracking are shown. **e** Cell fates of tracked structures. The types and numbers (in brackets) of embryogenic structures that were tracked is shown on the x-axis. The final cell fate of the embryogenic structures is shown as a percentage of the initial population on the y-axis. Statistical analysis was performed using a one-way ANOVA followed by a Student–Newman–Keuls test at the 95% confidence level. The error bars represent the standard error

Compact and loose callus are morphologically distinguished based on their larger cell size, increased cell wall thickness and early exine rupture, with compact callus showing partial exine rupture and loose callus showing complete exine rupture (Fig. 1a–h) (Li et al. 2014). The thickened cell walls of embryogenic callus can be seen after Renaissance SCR 2200 staining (Fig. 1e–h; Supplemental Fig. 3a). The cell walls of exine-enclosed embryos are thin and only weakly stained, the cell walls of embryogenic callus are thick and strongly stained, while the thickness of suspensor cell walls lies between these two extremes. Cell wall thickness can also be visualized indirectly with the Cell Brite membrane stain (Fig. 1i–l; Supplemental Fig. 3b). The plasma membranes of exine-enclosed embryo- and suspensor cells are in close proximity to each other, while large spaces can be seen between the plasma membranes of adjacent callus cells due to the thickened cell walls. Cell adhesion is maintained by the cell wall, and in particular by the pectin-rich middle lamella between individual cells. Embryogenic callus cells appear to lose their connectivity in regions where the surrounding exine has ruptured and separated from the underlying cell(s), leaving only a few thin fibers to connect these cells (Fig. 1m–o), while these fibers are more abundant between cells of exine-enclosed embryos (Fig. 1p–r). These fibers might be pectins, as reduced pectin staining was observed at the sites where the exine detached from callus- and suspensor cells (Fig. 1s–v). These data suggest that the reduced adhesion between callus cells is due in part to reduced pectin accumulation.

As shown previously (Li et al., 2014), compared to the HS treatment, both the initial proportion of embryogenic cells (compact exine-enclosed embryos and embryogenic callus) and the final differentiated embryo yield can be enhanced up to 10 times by a combined HS + 0.05 µM TSA treatment (Fig. 1w), but in both treatments only a small proportion of the initially embryogenic cells is able to form histodifferentiated embryos (Fig. 1x). Treatment with HS and a higher TSA concentration (HS + 0.5 µM TSA) induces at least 70% of the original donor cells toward embryogenesis (Fig. 1w), but compact exine-enclosed embryos and differentiated embryos are rarely formed (Fig. 1x).

### Time-lapse imaging system

We developed a time-lapse imaging system to follow the developmental fate of the different types of embryogenic structures observed in culture (Fig. 2). In this system, one to two layers of immature pollen were embedded in a thin layer of agarose in a gridded imaging chamber and covered with liquid medium (Fig. 2a). We adjusted the cell density, agarose concentration and thickness, the timing of immobilization, and the imaging parameters to obtain a time-lapse imaging protocol that maximized embryo development and yield, while allowing us to determine the fate of a large number of embryogenic structures. Microspore immobilization in agarose and subsequent imaging inhibited the development of embryogenic cells into histodifferentiated embryos to different degrees (Fig. 2b–d), as previously reported for other ME time-lapse imaging systems (Indrianto et al. 2001; de F. Maraschin et al. 2008; Daghma et al. 2012). We therefore immobilized cells at day 5 of culture, a time point that allowed us to morphologically identify the different embryogenic structures and to avoid major reductions in final embryo yield. Expression of the embryo-expressed *LEAFY COTYLEDON1* reporter (*LEC1:LEC1-GFP*) was used to further secure the initial identity and fate of the tracked structures. *LEC1* is expressed in both the embryo proper and suspensor, as well as in embryogenic callus (Lotan et al. 1998; Li et al. 2014) and therefore marks all the embryo structures observed in microspore embryo culture.

### Embryogenic callus develops into suspensor-bearing embryos

We determined the fate of embryogenic structures in haploid embryo culture by following the development of 369 *LEC1:LEC1-GFP*-positive cells from day 5 up to day 10 of culture (Fig. 2e). Microspore embryo cultures were induced by HS + 0.05 µM TSA to obtain sufficient embryogenic cells for tracking. Exine-enclosed embryos, loose and compact embryogenic callus, and suspensors/suspensor-bearing embryos were pre-selected for tracking.

**Fig. 3:**
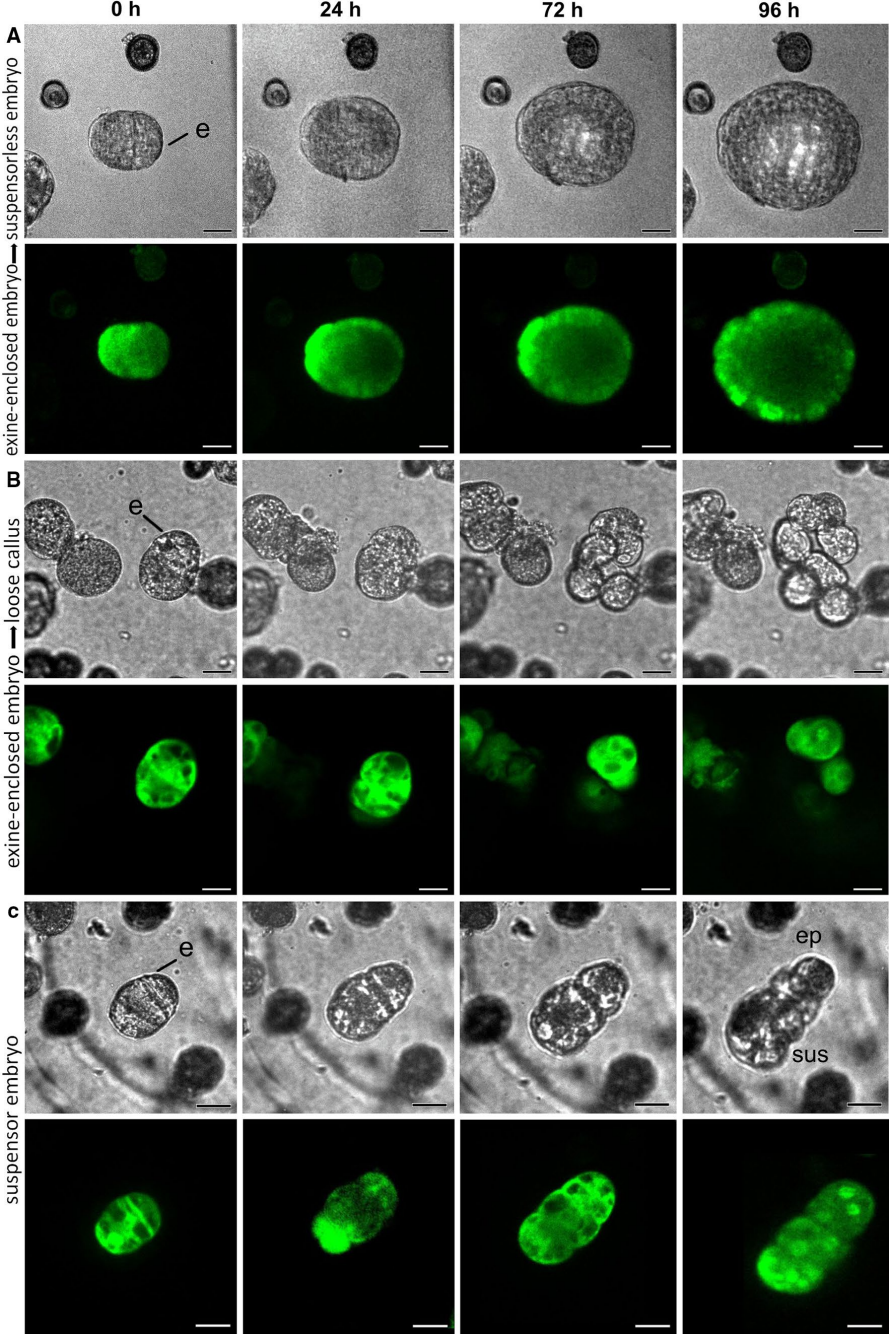
Developmental fate of exine-enclosed embryos and suspensor embryos. Cultures from *LEC1:LEC1-GFP* donor plants were immobilized on day five of culture and tracked for four days. For each set of panels, the light image is shown above and the CLSM image below. The green signal in the CLSM panels corresponds to LEC1-GFP expression. **a** An exine-enclosed embryo that develops into a suspensorless embryo. **b** An exine-enclosed embryo that develops into loose callus. **c** A few-celled suspensor embryo that continues to develop as a suspensor embryo. The suspensor can be distinguished from the embryo proper by the different division pattern of these cells; suspensor divisions are initially transverse, while the first embryo proper division is longitudinal. Labels: e, exine; sus, suspensor; ep, embryo proper

Compact exine-enclosed embryos were the major source of differentiated embryos, with ca. 44% of the tracked structures forming suspensorless embryos (Figs. 2e, 3a, Supplemental Table 1). The remaining exine-enclosed embryos either developed into embryogenic callus or showed little or no division during the tracking period (Figs. 2e, 3b). Regardless of their final fate, almost all compact exine-enclosed structures maintained their embryo identity during the culture period, as measured by the low proportion of cells that lost LEC1-GFP expression (Supplemental Table 1; Supplemental Fig. 4a).

**Fig. 4:**
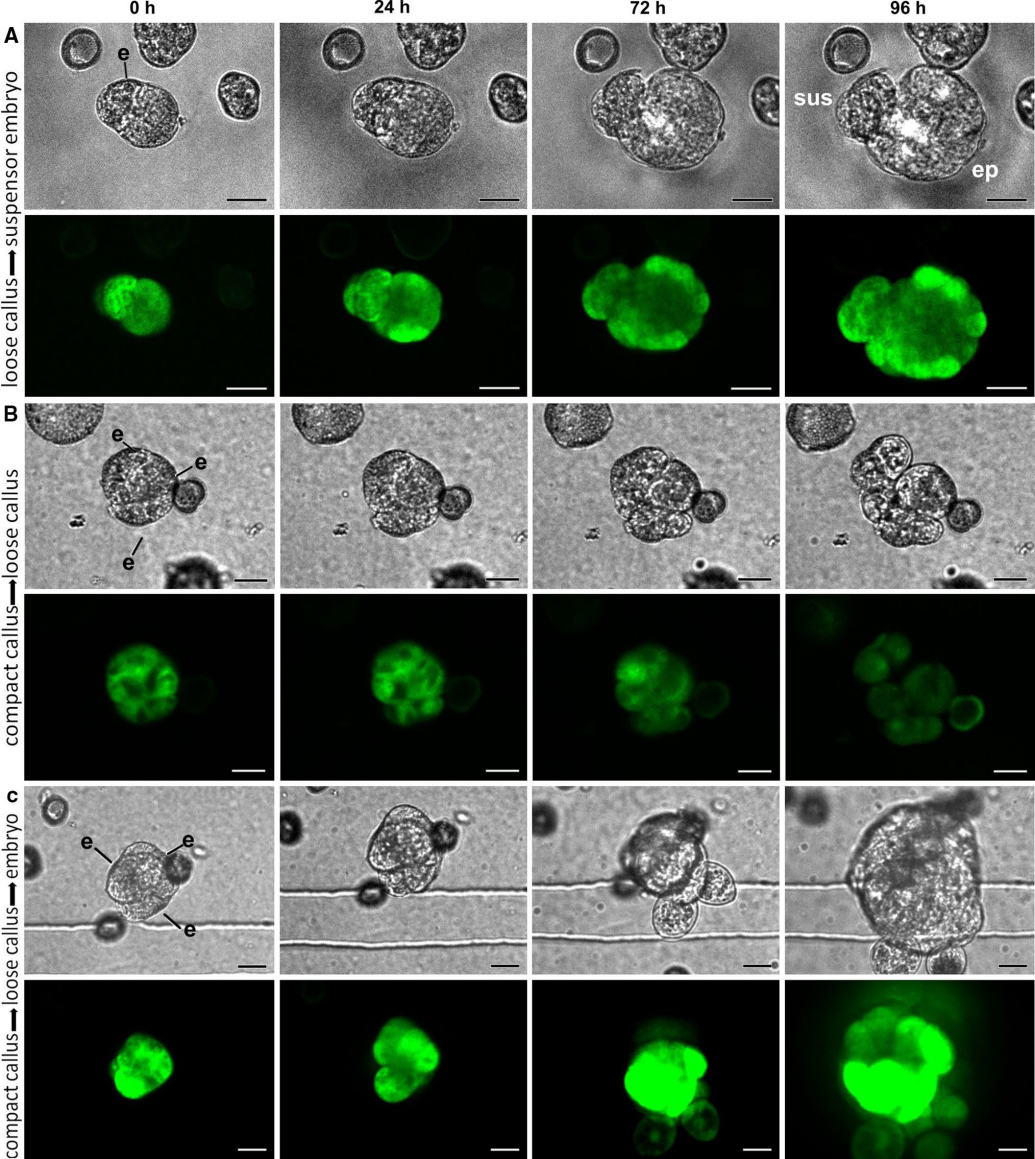
Developmental fate of embryogenic callus. Cultures from *LEC1:LEC1-GFP* donor plants were immobilized on day five of culture and tracked for four days. For each set of panels, the light image is shown on top and the CLSM image below. The green signal in the CLSM panels corresponds to LEC1-GFP expression. **a** loose callus that developed into a suspensor-bearing embryo. **b** Compact callus that developed into loose callus. **c**. Compact callus that developed into loose callus before becoming a suspensorless embryo

Of the 11 suspensors/suspensor-bearing embryos selected at the start of the tracking period, ca. 80% stopped dividing and the remaining 20% developed further as suspensor-bearing embryos (Figs. 2e, 3c, Supplemental Table 1). None of the tracked suspensor embryos differentiated into embryogenic callus. Despite their low growth potential, suspensors/suspensor embryos did not show an accompanying loss of LEC1-GFP expression (Supplemental Table 1). The loss of embryo identity and/or viability marked by decreased LEC1-GFP expression might occur later in these structures, i.e., beyond the tracking period.

As with suspensor embryos, ca. 80% of all embryogenic calli stopped dividing during the tracking period, suggesting a very low growth potential for these types of structures (Fig. 2e; Supplemental Table 1). This was also reflected in the relatively high proportion of callus cells that lost LEC1-GFP expression during the tracking period (Supplemental Table 1) and by the reduced viability of embryogenic callus (Supplemental Fig. 5). Of the two types of embryogenic callus found at the start of the tracking period, only loose callus contributed significantly to differentiated embryo yield and formed exclusively suspensor-bearing embryos from one or two cells of the callus (Figs. 2e, 4a; Supplemental Table 1). Of the compact callus structures that continued to divide, all formed loose callus, and of these, only one formed an embryo from a single, loosely connected cell (Figs. 2e, 4b, c; Supplemental Table 1). The large calli that developed as a result of cell tracking (Fig. 2d) appear to arise from globular embryos (Supplemental Fig. 4b).

The time-lapse imaging data highlight a number of key points. Firstly, the data support previous static cell biology studies that suggested that exine-enclosed embryos are the main progenitors of differentiated embryos, and that these embryos generally lack a suspensor. Secondly, unlike earlier studies (Telmer et al. 1995), we show that although the growth potential of embryogenic callus is very low, these structures are able to develop into suspensor-bearing embryos. The observation that loose callus rather than compact callus forms suspensor embryos is surprising, considering the poorer cell morphology of the former. Finally, suspensor-bearing embryos, whether they develop ab initio or from embryogenic callus, share the common feature of early exine rupture.

### Morphological parameters that define successful embryogenesis

**Fig. 5:**
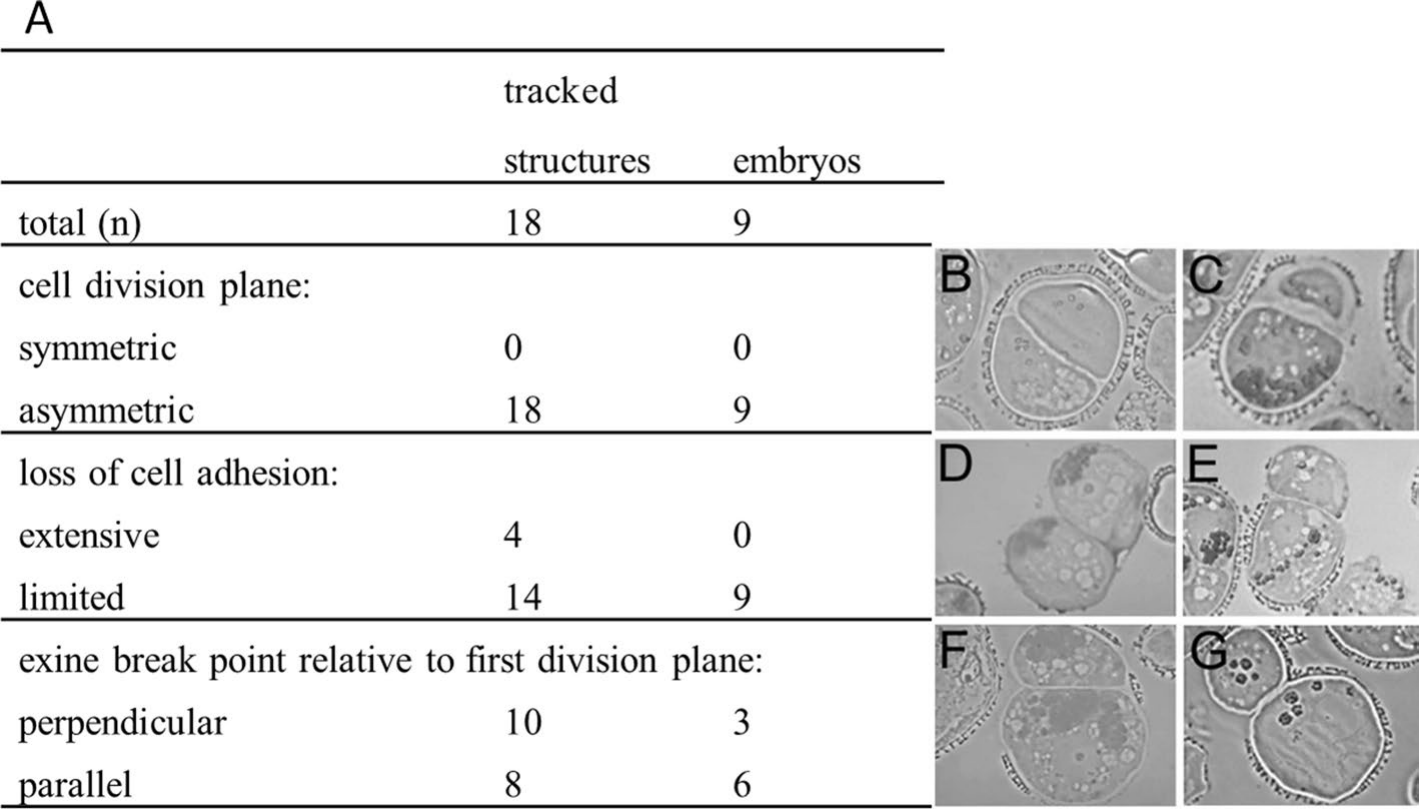
Key morphological characteristics of embryogenic callus structures that develop into suspensor-bearing embryos. **a** Table showing the number of tracked structures with the indicated initial morphological characteristics and their final cell fate. Cell morphology was defined at the start of the tracking period. Cultures were tracked for five days and then cultured without tracking for an additional four days, at which time the final cell fate was established. **b–f** Representative thin sections of the different callus morphologies. **b–c** Symmetric (**b**) or asymmetric (**c**) cell division plane. **d-e** Exine rupture perpendicular- (**d**) or parallel (**e**) to the division plane. **f-g** Limited (**f**) or extensive (**g**) loss of cell adhesion between two cells

Only a small proportion of the loose embryogenic callus that develops in microspore embryo culture is able to form a suspensor embryo. To understand why some loose callus clusters are able to form suspensor-bearing embryos, we analyzed the tracking images from 18 loose callus clusters with clearly defined morphological features at the start of the tracking period, nine of which developed into suspensor-bearing embryos. The initial cell division plane, the position of exine rupture, and the extent to which cell adhesion was lost were examined to determine whether any of these parameters is associated with successful embryo development (Fig. 5). In all cases, suspensor embryos were formed from loose callus cultures that initially comprised two to three cells. The data suggest that relatively limited exine rupture, relatively limited loss of cell adhesion, and an asymmetric first division are required for suspensor embryo formation. A fixed exine rupture plane, i.e., either parallel or perpendicular to the first cell division plane, was not a prerequisite for successful suspensor embryo formation; however, in all loose callus clusters that formed suspensor embryos, the exine still partially covered one the callus cells (Fig. 5c, e, f).

### Both embryo proper and suspensor identity is established ab initio in embryogenic callus

**Fig. 6:**
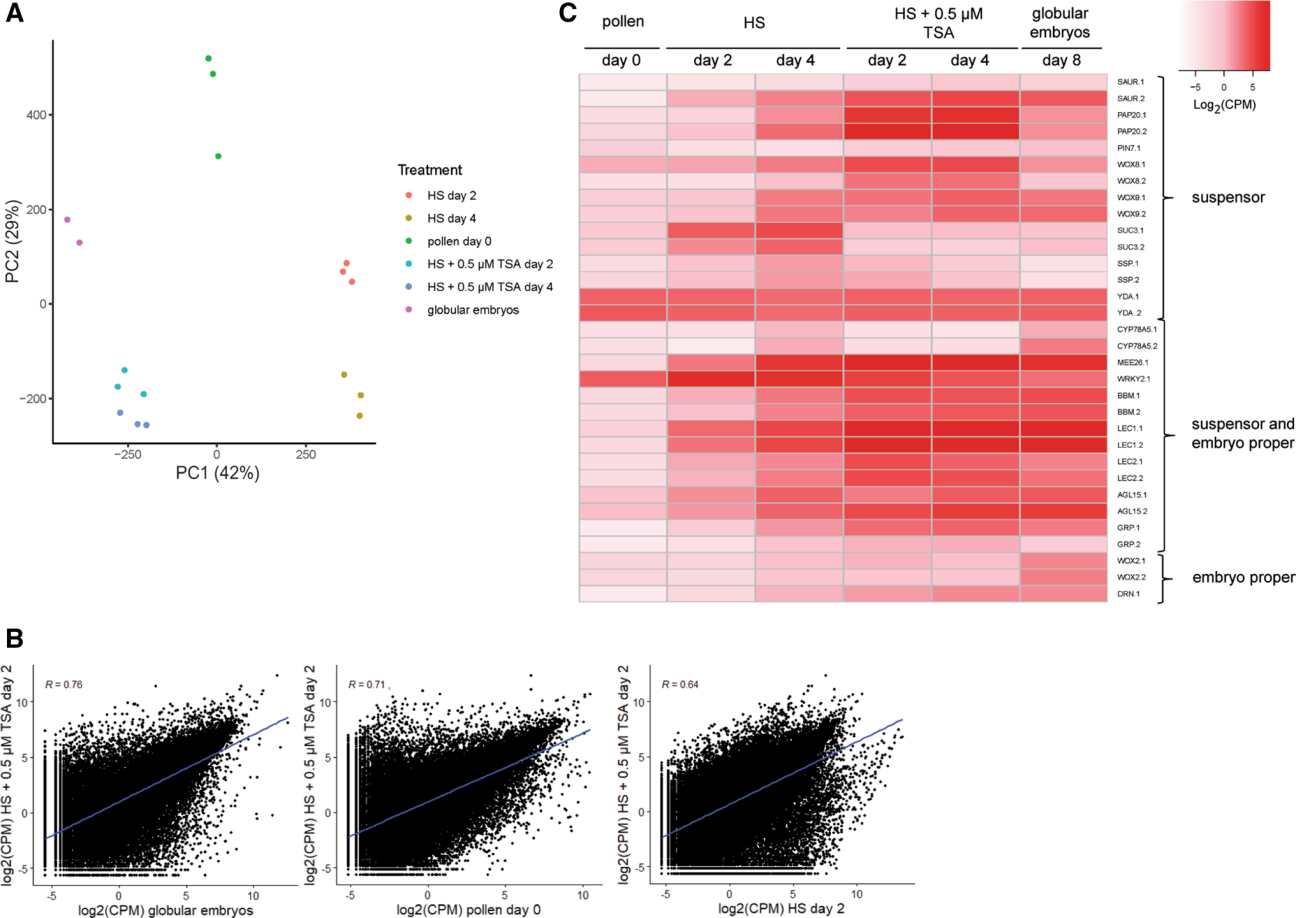
Expression profiles of *B. napus* embryo-expressed genes during pollen and haploid embryo development. **a** Principal component analysis (PCA) of the RNA-seq expression data for six different culture treatments. Biological replicates from the same treatment are represented by the same colour. **b** Scatterplots showing pairwise comparisons of gene expression data (log2CPM) from the indicated culture treatments. The Pearson correlation coefficients (R) and linear regression line are indicated. **c** Heatmap of RNA-Seq expression values (normalized log2(CPM)) for selected embryo-expressed genes in different cultures. The *Brassica napus* gene names and identifiers, as well as the relevant publications showing embryo or embryo region-enriched expression can be found in Supplemental Data File 1. Labels: d0, freshly isolated microspores/pollen; HS day 2, 2-day-old HS-treated cultures; HS day 4, 4-day-old HS-treated cultures; HS + 0.5 µM TSA day 2, 2-day-old HS + 0.5 µM TSA-treated cultures; HS + 0.5 µM TSA day 4, 4-day-old HS + 0.5 µM TSA-treated cultures; globular embryos, purified globular embryos from 8-day-old cultures treated with HS + 0.05 µM TSA

Our cell tracking data indicates that loose embryogenic callus forms suspensor embryos in culture, but it is not clear whether suspensor identity is already present in callus structures or whether it is acquired later. Suspensor- and embryo proper-expressed reporters such as *LEC1* and *GLYCINE-RICH PROTEIN* genes are expressed in few-celled loose and compact embryogenic callus (Soriano et al. 2014; Li et al. 2014), but we have not observed expression of the suspensor-expressed *PIN7:PIN7-GFP* reporter in embryogenic callus at this early stage of development (data not shown). To obtain a broader overview of embryogenic callus identity, we compared the transcriptomes of freshly isolated microspores/pollen, microspore/pollen treated with HS for two or four days to induce embryogenesis, and microspore/pollen treated with HS + 0.5 µM TSA for two or four days to induce a high proportion of embryogenic callus (Fig. 6; Fig. 1). At day 5 of culture HS cultures contain ca. 0.6% and 0.2%, embryogenic callus and exine-enclosed embryos, respectively, while HS + 0.5 µM TSA cultures contain ca. 65% and 5%, respectively (Fig. 1; Li et al. 2014). As a control, we included 8-day-old purified globular stage embryos that for the most part lack a defined suspensor. Principle component analysis (PCA) was used to analyze RNA-seq gene expression profiles of the different cultures. A plot of the samples on the first two principal components, together explaining 71% of the observed variability, is shown in Fig. 6a. The first principle component (PC1), which explains 42% of the variance, appears to describe the transition from a pollen-dominated gene expression state (HS) on one side of the axis to an embryo-dominated gene expression state (globular embryos and HS + 0.5 µM TSA) on the other side of the axis. In agreement with the PCA, Pearson correlation analysis (Fig. 6b) showed that the transcriptomes of the callus-enriched samples (HS + 0.5 µM TSA day 2) are more similar to that of globular-stage embryos than to cultures at day 0 or to heat-stressed cultures (HS day 2). These analyses suggest that callus is more embryo-like than pollen-like. Finally, to further characterize embryogenic callus, we examined whether individual genes whose expression is known to be enriched in the suspensor, the embryo proper or both are also expressed in embryogenic callus-enriched samples (Fig. 6c, Supplemental Data Set 1). The majority of genes in all three categories were not or only weakly expressed at the start of cultures, but in general their expression increased in HS cultures and even more so in HS + 0.5 µM TSA treated cultures, although the expression level of suspensor- and embryo-proper-enriched genes was generally lower than that of genes expressed in both domains. The expression of ‘suspensor-enriched’ genes in globular embryos, which generally lack a suspensor, most likely reflects expression of these genes in the basal embryo domain that differentiates after exine burst (Hause et al. 1994; Soriano et al. 2014). Among the suspensor-enriched genes, expression of *PIN7* and *CYP78A5* could not be detected in TSA-treated samples. *CYP78A5* was previously identified by microarray analysis as a suspensor-expressed gene in *B. napus* microspore embryo cultures*,* but the suspensors analyzed in this study were filamentous i.e. zygotic embryo-like (Joosen et al. 2007). Together the data suggest that both embryo and suspensor identity is initially induced in few-celled embryogenic calli, but that the expression of specific genes/cell identity pathways might be compromised in this system.

## Discussion

Time-lapse imaging is a valuable technique for understanding the cell fate decisions that take place during plant development. Following individual cells through the course of their development, either using light microscopy and/or fluorescent protein reporters that mark cell identity or subcellular processes, is an important first step in defining the mechanism driving these cell fate decisions (Heisler et al. 2005; Gooh et al. 2015; Kadokura et al. 2018; Kimata et al. 2019). Cultured plant cells are particularly well-suited to cell tracking, as they can be isolated in large numbers and can often be tracked from the single cell to the multicellular, differentiated stage. Time-lapse imaging has been used to follow the initial morphological changes that accompany microspore embryo development and have demonstrated that more complex developmental pathways can be defined than with static imaging alone (Indrianto et al. 2001; de F. Maraschin et al. 2005, 2008; Daghma et al. 2012, 2014). Here we used time-lapse imaging to determine the fate of three different types of embryogenic structures found in *B. napus* microspore embryo cultures: exine-enclosed embryos, suspensors/suspensor- bearing embryos, and compact and loose callus. In contrast to earlier time-lapse imaging studies, our study made use of multicellular structures rather than one-to-two cell gametophytes as the starting material, as this allowed us to clearly define the initial identity of the different types of embryogenic structures while maintaining a sufficiently high embryo yield during imaging. Improvements to the time-lapse imaging protocol, such as the use of liquid medium rather than agarose, should allow us to track a larger number of cells at an earlier time-point.

During *B. napus* zygotic embryogenesis, division of the zygote invariably generates an apical cell that develops into the embryo proper and a basal cell that generates the hypophysis (future root pole) and filamentous uniseriate suspensor (Tykarska 1976, 1979). By contrast, many more types of embryogenic development have been observed during *B. napus* ME. Exine-enclosed embryos are the most common type of embryogenic structures observed initially in culture. These structures grow within the confines of the exine until it can no longer restrain the growing embryo and bursts open. The majority of these embryos lack a suspensor. Our time-lapse imaging confirms earlier static cell biology observations in which exine-enclosed, embryos were proposed to be the main source of suspensorless embryos in *B. napus* microspore embryo culture.

Embryos with long uniseriate suspensors are also observed in microspore embryo cultures. These develop through a unique developmental pathway in which a filamentous suspensor develops first, followed by embryo proper development from the distal suspensor cell (Supena et al. 2008). This pathway is characterized by exine rupture as early as the two-cell stage. Embryos with more rudimentary types of suspensor development are also observed, but the developmental sequence leading to their formation  was not clear. Suspensor development is rarely observed in genotype DH12075, but when present is usually of the rudimentary type. Here we show that loose embryogenic callus, despite its low growth potential, is the main source of embryos with rudimentary suspensors in DH12075. It is surprising that loose callus structures form suspensor-bearing embryos, given their reduced intercellular connections. On the other hand, loose embryogenic callus cells are more similar to suspensor cells than embryo proper cells in terms of cell size, cell wall thickness and intercellular adhesion.

Early exine rupture appears to play a key role in promoting suspensor formation during *B. napus* microspore embryo culture. Tang et al. (2013) developed an elegant system in which the exine of heat-stressed microspores can be broken at one or more pollen germination furrows (so-called exine-dehisced microspores; EDM). Using this system, they showed that exine rupture parallel to the first division plane is required to establish apical (embryo proper) and basal (suspensor) embryo lineages in EDM, and that both symmetric and asymmetric divisions can generate suspensor embryos. Suspensor embryo formation from loose callus appears to be different from that in EDM in that in loose callus, only asymmetric cell divisions generated suspensor embryos and that exine rupture was not fixed to one division plane. In EDMs, the first cell division takes place after exine rupture and generates two cells with different morphologies, regardless whether the division was symmetric or asymmetric. The larger cell is cytoplasmically rich and forms the apical cell, while the smaller cell is vacuolate and forms the suspensor. Additional ultrastructural analyses should reveal whether callus cells that form suspensor embryos also show different morphologies.

Although early exine rupture in callus cells seems to be a prerequisite for establishment suspensor identity, the low growth potential of these cells suggest that other factors are required to commit callus cells to this developmental pathway. Filamentous suspensors express suspensor genes ab initio (Joosen et al. 2008; Soriano et al. 2014), while few-celled embryogenic callus cells appear to have a mixed identity, in that they express both suspensor-and embryo proper genes. This suggests that the initial commitment of these callus cells to either embryo proper or suspensor development is acquired later in development. During zygotic embryo development, the *DR5* auxin response reporter is expressed in the apical embryo proper in response to transport of auxin from the basal suspensor cells by PIN7 (Friml et al. 2003). During ME, *DR5* expression is observed in exine-enclosed embryos, but not in embryogenic callus (Soriano et al. 2014), despite the expression of other canonical embryo proper-enriched genes (*DRN*, *WOX2*) in these cell clusters. Likewise, *PIN7* was not expressed in embryogenic callus, despite expression of other suspensor-expressed genes (*WOX8*, *WOX9*). In this respect, the lack of a *DR5* response and *PIN7* expression might indicate that the polar auxin transport mechanism required to fix separate embryo proper and suspensor cell fates (Friml et al. 2003; Blilou et al. 2005) is (initially) missing in embryogenic callus.

Why does embryogenic callus initially develop in microspore embryo cultures? One clue lies in the observation that microspores/pollen cultured under less extreme temperature regimes have a higher potential to develop into suspensors/suspensor-bearing embryos (Supena et al. 2008). Prem et al. (2012) also showed that microspores and pollen cultured for a long period under non-embryo inductive conditions (18 °C) develop into suspensor-bearing embryos. Notably, these cells resemble embryogenic callus in that they show early exine rupture, reduced adhesion, and thickened cell walls. The milder inducer treatments used to induce suspensor embryo formation suggest that the donor cells are less competent to respond to more intense stress regimes. Treatment of microspore cultures with HS + TSA induces a higher proportion of differentiated embryos, but also proportionately more embryogenic callus and suspensor-bearing embryos. TSA has been proposed to amplify the protein acetylation process that normally occurs in response to HS. The HS + TSA treatment could therefore be seen as a more severe form of abiotic stress. However, although the HS + TSA treatment triggers more cells to acquire embryo identity, many of these cells might not be fully competent to respond/adapt to this stress, and develop into embryogenic callus/suspensor embryos, rather than exine-enclosed embryos.

### Author contribution statement

PC-M, CS, and AH performed experiments and analyzed data. All authors designed experiments. PC-M and KB wrote the paper with input from the other authors.

## Electronic supplementary material

Below is the link to the electronic supplementary material.

Supplementary file1 (XLSX 16 kb)

Supplementary file2 (PDF 487 kb)

## Abbreviations

CLSM: Confocal laser scanning microscopy
FESEM: Field emission scanning electron microscopy
HS: Heat stress
LEC1: LEAFY COTYLEDON1
Microspore embryogenesis: ME
TSA: Trichostatin A
