# Supplementary material for: Live Imaging of embryogenic structures in Brassica napus microspore embryo cultures highlights the developmental plasticity of induced totipotent cells
Source: Plant Reprod. 2020 Jul 10;33(3):143–58. doi: 10.1007/s00497-020-00391-z (PMC7648746; doi:10.1007/s00497-020-00391-z)
Supplement: Supplementary file 2 — Supplementary file2 (PDF 487 kb) [file 497_2020_391_MOESM2_ESM.pdf]

Supplemental Data

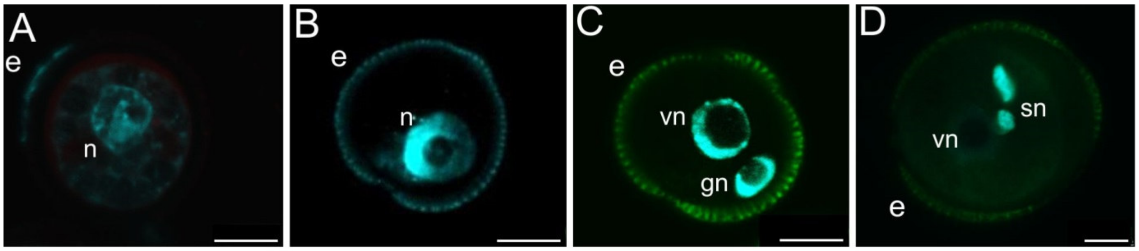

Supplemental Figure 1. Male gametophyte development.

CLSM images of DAPI (blue)-stained microspores and pollen.

A. mid-uninucleate microspore with a centrally located nucleus (n).

B. late uninucleate microspore with the nucleus (n) close to one of the lobes.

C. binucleate pollen grain with a vegetative nucleus (vn) and a smaller generative nucleus (gn).

D. trinucleate pollen grain with a vegetative nucleus and two sperm nuclei (sn). The exine (e) autofluoresces in the DAPI channel.

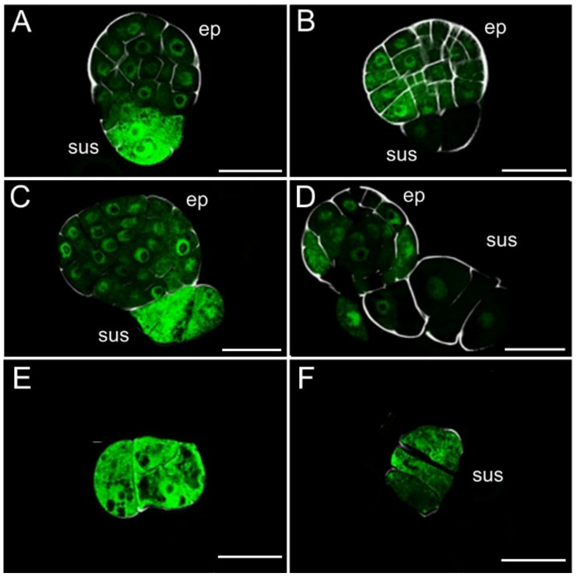

Supplemental Figure 2. Suspensor morphologies observed in culture.

A. Suspensor-bearing embryo where the suspensor is a small protrusion.

B. Suspensor-bearing embryo where a suspensor cell divided longitudinally.

C. Suspensor-bearing embryo where the suspensor shows oblique division planes.

D. Suspensor-bearing embryo where the suspensor shows oblique division planes and large cells.

E. Few-celled suspensor-bearing embryo where the suspensor shows oblique cell divisions.

F. A suspensor with transverse cell divisions. No embryo proper is visible.

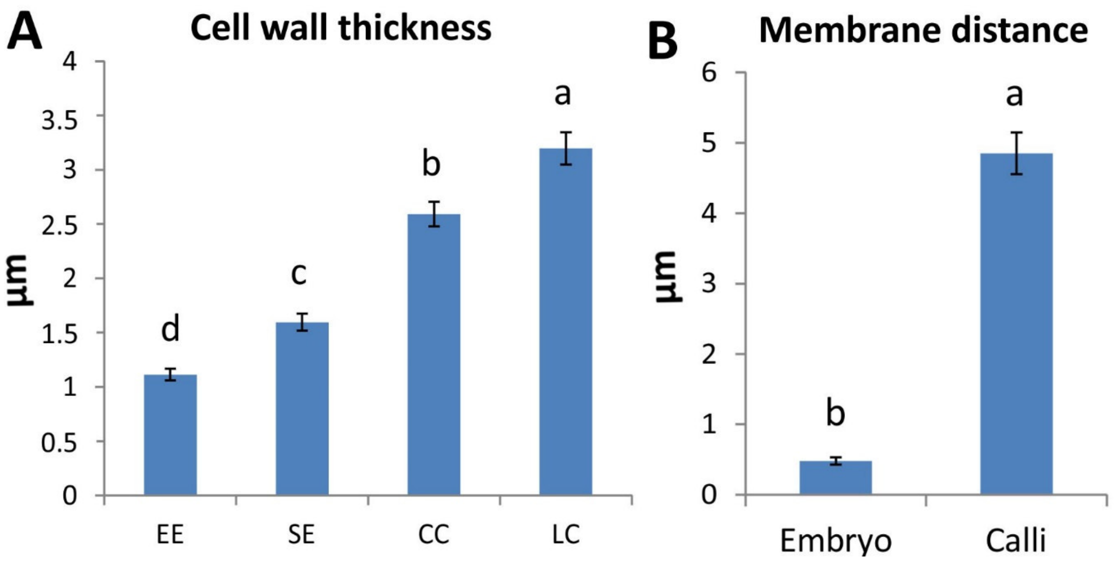

Supplemental Figure 3. Cell wall thickness and distance between membranes in the different types of embryogenic structures found in five-day-old HS + 0.05 uM TSA-treated cultures

A. Cell wall thickness in the different embryogenic structures. EE, exine-enclosed embryos; SE, suspensors/suspensor embryos; CC, compact callus; LC, loose callus.

B. Distance between adjacent plasma membranes in embryogenic structures. Embryo, exine-enclosed embryos and suspensor embryos. Calli, loose and compact callus.

Statistically significant differences in cell wall thickness and distance between adjacent membranes in different categories was calculated using Mood's median non-parametric test at $p < 0.\ 05$. Significantly different values are assigned different letters above the plot.

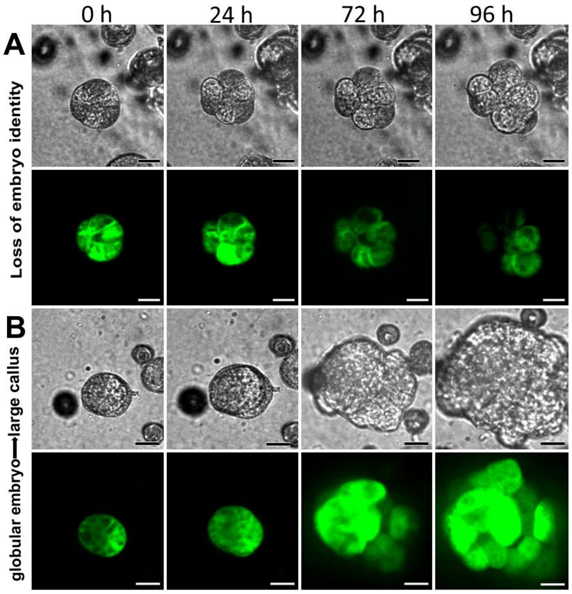

Supplemental Figure 4. Additional cell fates observed in microspore embryo cultures

Cultures from *LEC1:LEC1-GFP* donor plants were immobilized on the fifth day of culture and tracked for four days. For each set of panels, the light image is shown on top and the CLSM image below. LEC1-GFP is shown in green.

A. Loss of embryo identity, as visualized by reduced LEC1-GFP expression in an exine-enclosed embryo as it develops into loose callus.

B. The large callus-like structures observed only in immobilized and tracked cultures originate from exine-enclosed embryos.

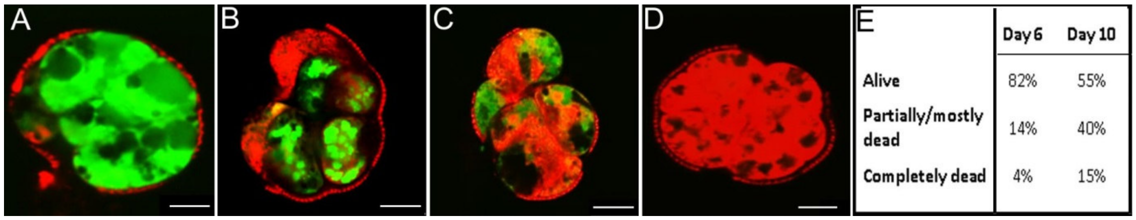

E

	Day 6	Day 10
Alive	82%	55%
Partially/mostly dead	14%	40%
Completely dead	4%	15%

Supplemental Figure 5. Cell viability in different types of embryogenic structures.

Representative live whole-mount samples from six-day-old cultures stained with fluorescein diacetate (FDA) and propidium iodide (PI). Live cells take up the FDA stain (green). PI is localized to the plasma membrane of live cells, and to the cytoplasm of dead cells,

A. Exine-enclosed structures.

B. Callus-like structures in which some of the cells are dead.

C. Callus-like structures in which the majority of cells are dead.

D. Callus-like structures in which all cells are dead.

E. The proportion of multicellular embryogenic structures at day 6 and 10 of culture that are viable or show partial- or complete cell death.

Supplemental Table 1. Developmental fates of tracked embryogenic structures

	arrested or limited cell divisions (%)				sustained cell divisions (%)		
**embryogenic structures (# tracked)**	**arrested**	**limited cell divisions**	**arrested with loss of GFP expression**	**total**	**embryos formed without suspensor**	**embryos formed with suspensor**	**callus**
exine-enclosed (101)	19.9	14	1.0	34.9	44.4	0	20.7
suspensor/suspensor embryo (11)	36.4	36.4	0	72.8	0	27.2	0
compact callus (197)	20.8	34	22.8	77.6	0.5	0	21.9
loose callus (60)	14.7	38.6	29.3	82.6	0	10	7.4
